# First insights into a type II toxin-antitoxin system from the clinical isolate *Mycobacterium* sp. MHSD3, similar to epsilon/zeta systems

**DOI:** 10.1371/journal.pone.0189459

**Published:** 2017-12-13

**Authors:** Daniel Jaén-Luchoro, Francisco Aliaga-Lozano, Rosa Maria Gomila, Margarita Gomila, Francisco Salvà-Serra, Jorge Lalucat, Antoni Bennasar-Figueras

**Affiliations:** 1 Microbiologia, Departament de Biologia, Universitat de les Illes Balears, Palma de Mallorca, Spain; 2 Department of Infectious Diseases, Sahlgrenska Academy, University of Gothenburg, Gothenburg, Sweden; 3 Centre for Antibiotic Resistance Research (CARe) at University of Gothenburg, Gothenburg, Sweden; 4 Laboratorio de Biología Molecular, Clínica Rotger, Palma de Mallorca, Spain; 5 Serveis Cientifico-Tècnics, Universitat de les Illes Balears, Palma de Mallorca, Spain; 6 Institut Mediterrani d’Estudis Avançats (IMEDEA, CSIC-UIB), Esporles, Spain; 7 Area de Enfermedades Infecciosas, Instituto Universitario de Investigaciones en Ciencias de la Salud (IUNICS-UIB), Universitat de les Illes Balears, Palma de Mallorca, Spain; Karl-Franzens-Universitat Graz, AUSTRIA

## Abstract

A putative type II toxin-antitoxin (TA) system was found in the clinical isolate *Mycobacterium* sp. MHSD3, a strain closely related to *Mycobacterium chelonae*. Further analyses of the protein sequences of the two genes revealed the presence of domains related to a TA system. BLAST analyses indicated the presence of closely related proteins in the genomes of other recently published *M*. *chelonae* strains. The functionality of both elements of the TA system was demonstrated when expressed in *Escherichia coli* cells, and the predicted structure of the toxin is very similar to those of well-known zeta-toxins, leading to the definition of a type II TA system similar to epsilon/zeta TA systems in strains that are closely related to *M*. *chelonae*.

## Introduction

Toxin-antitoxin (TA) systems are small genetic elements that encode a toxin and its cognate antitoxin. These two elements are expressed and interact with each other, establishing a situation in which the antitoxin blocks the effect of the toxin. In many cases, under conditions of stress, the lower stability of the antitoxin promotes faster degradation, allowing the toxin to exert its effect [1]. TA systems are widely distributed among bacteria and archaea [2]. They are classified as type I when the toxin is inhibited by antisense RNAs at the mRNA level [3]; type II when both elements are translated into a protein and the inhibition is caused by the protein-protein interaction [4]; type III when the antitoxin is a small RNA that inhibits the toxin’s effect by directly interacting with the protein [5]; type IV, designated for the *yee*U/*yee*V TA system of *Escherichia coli*, in which the regulation implies the stabilization of other proteins [6]; and type V, designated for the recently described ghoS/ghoT TA system found in *E*. *coli*, where the antitoxin acts as a ribonuclease that degrades the mRNA of the toxin, inhibiting its translation [7]. The biological function of TA systems has been linked to the protection of mobile genetic elements, formation of bacterial persistence, and survival under conditions of stress, among other presumptive functions [8–11].

The epsilon/zeta loci are widespread type II TA systems found among both Gram-positive and negative bacteria [12]. They were first discovered in the plasmid pSM19035 of *Streptococcus pyogenes* [13,14], Further studies showed that these two genes are crucial to ensuring the maintenance of the plasmid [13,15]. It is also known that epsilon/zeta systems are encoded by the bacterial chromosome associated with integrative or conjugative genetic elements [16–18]. The first chromosomally encoded epsilon/zeta system described was the PezAT system of *Streptococcus* spp. [18].

Maintenance of mobile genetic elements can be related to plasmid encoded systems, which act as an addiction module that only allows the survival of the members of the population that preserve the plasmid during cell division. Loss of the toxin-antitoxin system, for example through loss of the plasmid, along with higher instability of the antitoxin leads to cell death in a process called postsegregational killing (PSK) [19]. Regarding to the chromosomally encoded epsilon/zeta systems, a good example are the epsilon/zeta systems found in pneumococcal pathogenicity islands, where they seem to have an influence in the progression of pneumococcal infections, acting as a possible virulence factor [20,21].

The epsilon/zeta systems were first determined to directly target cell wall formation [20] and can cause either a bactericide effect or growth arrest [15], acting in an ATP dependent manner [18,19]. Moreover, two different mechanisms of action for the epsilon/zeta systems have been described so far. In the first one, the cell wall formation is affected through phosphorylation of the UDP-N-acetylglucosamine (UNAG) on its 3’-hydroxyl group [20,22], causing a reversible growth arrest [23,24]. The second mechanism of action implies the phosphorylation of nicotinamide adenine dinucleotide (NAD) or its precursor, nicotinic acid adenine dinucleotide (NAAD), at the adenosine 3’-hydroxil group, affecting NAD/NAAD-dependent pathways [25]. This mechanism was described in the system AvrRxo1-AvrRxo2 of the plant pathogen *Xanthomonas oryzae* pv. *oryzicola* and was shown to cause reversible dormancy when expressed in *E*. *coli* [26]. Plasmid-encoded TA systems (including epsilon/zeta) seem to mainly guarantee the stable inheritance of plasmids, and consequently the maintenance of the extrachromosomal genetic information; otherwise, the plasmid-bearing bacterial populations would be metabolically handicapped in comparison to plasmid-free cells, for example, when competing for establishment in the host environment. On the other hand, the chromosomally encoded TA homologues would contribute to maintenance of the bacteria in the host [21]. For this reason and in the context of pathogenicity, chromosomal toxin-antitoxin systems seem to be designed to be more bacteriostatic rather than bactericidal since they assure competitiveness and cell survival in host.

Due to their potential influence on pathogenicity, it is important to consider the presence of epsilon/zeta TA systems as well as other types of TA systems in species with clinical relevance because they can be considered to be targets for the development of new treatments [4] that can improve a patient’s prognosis, especially in hard-to-treat infections, such as those caused by members of the *Mycobacterium* genus. Most of these TA systems have been described in *M*. *tuberculosis* [27]. TA systems have also been detected in rapid growing mycobacteria (RGM), such as *Mycobacterium smegmatis*, the inactivation of which demonstrated that it affects survival of the bacteria [28]. This effect in *M*. *smegmatis* could be important for the treatment of infections caused by other RGM, considering relevant emerging opportunistic pathogens, such as *Mycobacterium chelonae*, which is commonly responsible for skin infections, as well as reported cases of soft-tissue and bone infections or infections due to prosthetic joints or transplants [29–33].

Here, we describe a TA system found in the clinical strain *Mycobacterium* sp. MHSD3 isolated in a previous study [34], which is closely related to *M*. *chelonae*. A better understanding of this TA system will be useful to better understand the resources available for opportunistic pathogenic mycobacteria and their use as potential targets for the treatment of hard-to-treat infections.

## Materials and methods

### Strain isolation, growth conditions, DNA isolation and sequencing

*Mycobacterium* sp. strain MHSD3 was isolated from a human tissue biopsy in Son Dureta University Hospital (Mallorca, Spain) [34]. Strain MHSD3 was cultured on R2A agar plates at 30 ºC for 4 days. Cells were pretreated with Buffer ATL (Qiagen, Hilden, Germany) and proteinase K at 56 ºC for 1 h. Mechanical disruption was applied using a DisruptorGenie® (Scientific Industries, Bohemia, New York, United States) for 5 min. DNA extraction was performed with a Wizard® Genomic DNA Purification Kit (Promega, Madison, Wisconsin, United States), and the resulting sample was purified with the DNA Clean & Concentrator^TM^-100 Kit (Zymoresearch, Irvine, California, United States). DNA was sequenced on an Illumina HiSeq 2500 platform for Illumina Paired-End reads (2x100 cycles paired-end, insert size 250–350±50). Raw reads were filtered with Sickle v1.33 [35]. A *de novo* assembly was constructed using Velvet v1.1.06 [36] with 50x coverage of high-quality Illumina reads. The resulting assembly was improved using the Post-Assembly Genome Improvement Toolkit (PAGIT) [37] using the genome of the type strain *M*. *chelonae* CCUG 47445^T^ (CP007220) as a reference. Gaps were filled using GapFiller v1.10 [38] and remaining indeterminants were eliminated using in-house Perl scripts to assemble contigs. The genome was annotated with Prokka v1.10 [39] for in-house work and was deposited in GenBank with PGAP annotation [40].

### Identification of the toxin-antitoxin (TA) system

TA systems were searched for in the *Mycobacterium* sp. MHSD3 genome after annotation with Prokka v1.10 and searching the Toxin-Antitoxin DataBase (TADB) [41]. The protein sequences identified as potential TA system members were analyzed using Pfam [42] and UniProt [43] to identify homologous proteins in other genomes by BLAST using a threshold of at least 50% identity covering at least 50% of the sequence. Sequences of the closely related proteins were aligned with ClustalO v1.2.1 [44] with 10 iterations. A maximum-likelihood-based tree was performed with 100 bootstraps using PhyML v3.0 [45].

### TA system PCR amplification

TA system genes were amplified through 35 PCR cycles (denaturation at 95°C, 1 min; annealing at 55°C, 1 min; elongation at 72°C, 1 min 30 sec) that included an initial denaturation step at 95°C for 10 min and a final elongation step at 72°C for 10 min. Each PCR was prepared with a final concentration of 1x PCR buffer (BIORON, Ludwigshafen, Germany), 0.2 mM dNTPs (0.05 mM each) and 2.5 U DFS-Taq Polymerase (BIORON). Reactions were adjusted with milli-Q water to a final volume of 50 μl. PCR products were purified using the Illustra™ GFX™ PCR DNA and Gel Band Purification Kit (GE Healthcare, Chicago, Illinois, United States), and the sequence was confirmed using the BigDye® Terminator v3.1 Cycle Sequencing Kit (version 3.1) and an automatic 3130 Genetic Analyzer DNA sequencer (Applied Biosystems, Foster City, California, United States) platform. The primers used are listed in Table 1.

**Table 1:** List of primers used in the amplification of the fragments and the confirmation of the clones. Restriction sequence included in the forward (*Nco*I) and reverse (*Hind*III) primers are indicated in bold.

Target	Primer F (5'→3')	Primer R (5'→3')	Reference
**Zeta-toxin**	TACCCA**CCATGG**TGAAACGGCTCGATCTGATCGTC	TTTGAC**AAGCTT**TACTCACACATCGGACGCTA	This work
**Hypothetical protein**	GATATC**CCATGG**CGGCTCCGGTAGA	TTGGGA**AAGCTT**ATCAGATCGAGCCGTTTCAC	This work
**pBAD/HA**	ATGCCATAGCATTTTTATCC	---	Invitrogen
**pRSF-Duet (MCS1)**	GGATCTCGACGCTCTCCCT	---	Merck Millipore

### Molecular cloning

Two expression vectors were used: i) pBAD/HA (Invitrogen, Carlsbad, California, United States), which contains the P_*BAD*_ promoter that is inducible in the presence of arabinose and carries the ampicillin resistance gene as a selection marker, and ii) pRSF-Duet^TM^ (Merck Millipore, Darmstadt, Germany), which contains the T7 promoter with the lac operator, is inducible with IPTG and carries a kanamycin resistance gene as a selection marker. The antitoxin component of the TA system was cloned into pBAD/HA and the toxin component into pRSF-Duet^TM^. Vectors and amplicons were double-digested with *Nco*I and *Hind*III (TaKaRa, Kusatsu, Shiga, Japan) through two successive reactions at 37 ºC for 3 h following the manufacturer's recommendations. After each digestion, the products were purified with the Illustra^TM^ GFX^TM^ PCR DNA and Gel Band Purification Kit (GE Healthcare, Chicago, Illinois, United States). Ligations were performed with T4 Ligase (TaKaRa) in a final volume of 20 μl according to the manufacturer's recommendations. A molar ratio of 1:3 (vector:insert) was used for the ligations.

Two microliters of each ligation were used to simultaneously transform chemically competent *E*. *coli* BL21 (DE3) pLysS (Invitrogen) cells following the manufacturer’s instructions. The cells were cultivated in Luria Bertani (LB) agar plates supplemented with 30 μg/ml kanamycin (Km), 50 μg/ml ampicillin (Ap) and 34 μg/ml chloramphenicol (Cm, for pLysS selection; the plasmid was already included in the strain). Plates were incubated at 37°C for 16 h.

Clones with the expected phenotype for each of the three plasmids were incubated in LB broth for 12 h with orbital shaking (180 rpm) at 37°C to maintain the antibiotic selective pressure. Plasmids were extracted using the UltraClean® 6 Minute Mini Plasmid Prep Kit (Mo Bio laboratories, Carlsbad, California, United States). Clones were analyzed by Sanger sequencing to verify whether the inserts were cloned in the correct open reading frame and orientation by PCR amplification using a plasmid-based forward primer (Table 1) and the reverse primer used to generate the original inserts.

### TA system experimental assay

A modified TA system test was performed according to a previously described protocol [46]. Pre-inoculum of a previously confirmed clone was made in 5 ml of LB broth supplemented with Km (30 μg/ml), Ap (50 μg/ml) and Cm (34 μg/ml) and incubated overnight with orbital shaking (180 rpm) at 37°C. Four Erlenmeyer flasks containing 50 ml of sterile LB broth supplemented with antibiotics were independently inoculated with 100 μl of pre-inoculum and incubated at 37°C with shaking at 180 rpm for 3 h until the culture reached the starting exponential phase. The TA system was then induced using four different conditions (one per culture): (1) control (without induction), (2) induction of the antitoxin by supplementing 0.3% (w/v) arabinose, (3) induction of the toxin by supplementing 0.2 mM IPTG, and (4) induction of both elements (0.3% (w/v) arabinose and 0.2 mM IPTG). The experiment was maintained for 7 h until the culture reached the stationary phase. Optical density was measured (1 ml sample from each culture) at 30-min intervals on an Ultrospec 100 Pro Spectrophotometer (GE Healthcare, Chicago, Illinois, United States). After induction, the number of CFU/ml was determined (in duplicate) from LB agar plates supplemented with 30 μg/ml Km, 50 μg/ml Ap, 34 μg/ml Cm and arabinose 0.3% (w/v) to protect cells from the toxic effects of the remaining toxin inside the cells. Plates were incubated at 37°C overnight.

### Analysis of protein expression

Cells from 7-h cultures were recovered from 50-ml samples that were centrifuged at 6,000 x g for 4 min. The pellet was washed with 1 ml of 50 mM Tris, pH 8.0, and resuspended in 800 μl of the same buffer. Cells were disrupted by sonication (pulses of 5 seconds, followed by 10 seconds of rest for 2 min). The samples were centrifuged at 3,000 x g for 2 min and the supernatant was centrifuged again for 30 min at 16,000 x g.

Supernatants (total volume 20 μl) were loaded onto 4–20% (w/v) polyacrylamide gels for electrophoresis in a ready-made Amersham ECL Gel (GE Healthcare) using the ECL Gel Box (GE Healthcare, Chicago, Illinois, United States) with the Precision Plus Protein^TM^ Dual Xtra Marker (2 to 250 kDa) (Bio-Rad, Hercules, California, United States). Gels were stained overnight with Coomassie Brilliant Blue and destained with a solution of 5% (v/v) ethanol and 7.5% (v/v) acetic acid at 55°C for 2 h.

### Protein identification

Using the annotated sequences, the expected mass of the desired proteins was predicted using the pI/Mw tool (http://web.expasy.org/). Bands of the expected mass were excised from the gel with a clean scalpel. Each gel slice was cut into smaller pieces and the pieces transferred to a sterile 1.5-ml Eppendorf tube. One hundred microliters of 400 mM NH_4_HCO_3_ and 100 μl of CH_3_CN were added and mixed by vortexing for 20 min. The supernatant was removed, and the process repeated. The samples were washed twice with 100 μl CH_3_CN, incubated for 10 min after each wash, and the supernatant was removed and the gel band dried at room temperature. Samples were kept on ice. The gel band was rehydrated with 50 μl of a pre-chilled trypsin solution (20 μg/ml in 50 mM NH_4_HCO_3_), incubated at 4°C for 30 min and at 37°C overnight. Five microliters of 5% (v/v) formic acid were added and mixed by vortexing for 5 min. Finally, 30 μl of the extraction solution (250 μl of CH_3_CN, 25 μl of trifluoroacetic acid and 225 μl of H_2_O) was added to the mixture. The extraction process was repeated with 30 μl of the extraction solution. The two extracts were pooled together, and 1 μl of the extract was deposited onto a MALDI-TOF MS plate (polished-steel, Bruker Daltonics, Billerica, Massachusetts, United States) and dried at room temperature. One microliter of matrix (saturated solution of 2,5-hydroxybenzoic acid or α-cyano-4-hydroxy-cinnamic acid in 70:30 acetonitrile:water with 0.1% trifluoracetic acid) was added and dried at room temperature. The sample was then analyzed on an Autoflex III MALDI-TOF-TOF (Bruker Daltonics, Billerica, Massachusetts, United States) spectrometer using Compassflex series v1.4 (flexControl v3.4, flexAnalysis v3.4 and BioTools 3.2). The spectra were calibrated using the Peptide Calibration Standard (Bruker Daltonics, Billerica, Massachusetts, United States). The obtained mass spectra were used for protein identification in the Swiss-Prot database and an in-house database created with the predicted protein sequences of the toxins and antitoxins found in the sequenced genome. The search process was performed with the algorithm Mascot (Matrix Sciences Ltd., http://www.matrixscience.com).

### Protein structure prediction

The amino acid sequence of each protein of the TA system was used to predict the structure and binding sites with I-TASSER [47–49]. The features, best model and best structural analogs were selected by the C-score, which ranged between -5 and 2 (higher values indicate a higher confidence in the model obtained), and the TM-score, which measures the structural similarity between two proteins, with values ranging between 0 and 1, with 1 indicating a perfect structural match [50]. I-TASSER uses the structures available in the Worldwide Protein Data Bank (wwPDB) for predictions. Alignment of the structurally similar proteins was performed with partial order structure alignment (POSA) [51].

## Results

### Assembly and annotation

Illumina sequencing of the strain produced 3,362,577 (x2) reads with an average length of 126 nucleotides and a mean Phred quality score of 34. The total yield was 846 Mb. The assembly resulted in 70 contigs with a 64.12% G-C content and total length of 4,939,932 bp. The N50 was 141,658 bp, and the largest contig was 361,417 bp. Annotation with Prokka v1.10 revealed 4,909 genes, 4,862 CDSs and 43 tRNAs. One complete ribosomal operon was found, and no plasmid elements were detected. The genome draft was deposited after annotation with PGAP into GenBank (http://www.ncbi.nlm.nih.gov) under accession number NADK00000000.

### Identification of the TA system and sequence characterization

The annotation revealed the presence of a putative zeta-toxin preceded by a gene that was annotated as a hypothetical protein (H.P.) (ATPase and H.P. with locus tag B5566_13860 and B5566_13855 in PGAP annotation, respectively). Protein sequence analysis of both genes with was performed with Pfam (Table 2). The H.P. was assigned to the superfamily MetJ/Arc repressors, which includes 35 families of proteins that carry ribbon-helix-helix DNA-binding motif, including several families of type II antitoxins. According to Pfam, the H.P. contained a domain similar to the ParD-like antitoxin family (Pfam code PF11903). The domain search results were obtained by using 56 consecutive amino acids out of 119 amino acids of the entire sequence, meaning that almost 53% of the sequences were not used by Pfam analysis. The H.P. and the ParD antitoxin of *E*. *coli* (UniProt accession number P22995) shared a 44% of identity in only 5% of the sequence. Evidently, these results do not allow a classification of the H.P. in any specific family of antitoxins according to Pfam, but are revealing a potential relationship with already described antitoxins. The toxin was assigned to the P-loop containing nucleoside triphosphate hydrolase superfamily (CL0023), which is composed by 217 families. Moreover, Pfam detected a domain similar to the one present in the AAA_33 family (Pfam entry PF13671), a family formed by members by far longer than the toxin but containing an ATP-binding motif. This assignment was based on the 65% of the sequence (127 out of 195 amino acids).

**Table 2:** Results of the domain search with Pfam, using the amino acid sequence of each protein.

	Length (aa)	Family	Clan	Entry type	Alignment	HMM	HMM length	Bit-score	E-Value
Zeta toxin	195	AAA_33	CL0023	domain	6–132	3–138	143	55.3	7.2E-15
H.P.	119	ParD like	CL0057	domain	7–62	2–57	73	62.4	2.9E-17

Sequence based comparisons were performed against the respective antitoxins and toxins of the TA systems pezAT of *Streptococcus pneumoniae* ATCC BAA-334 (UniProt accession numbers Q97QZ2 and Q97QZ1), ε-ζ of the plasmid pSM19035 of *S*. *pyogenes* (UniProt accession numbers Q57231 and Q54944) and the system AvrRxo1-AvrRxo2 of *X*. *oryzae* pv. *oryzicola* (UniProt accession numbers Q6TKR9 and Q6TKR8). BLAST analysis for the H.P. showed only 33% in the 17% of the sequence of PezA (Table A in S4 Table). The zeta toxin showed highest results against the ζ-toxin of *S*. *pyogenes*, with a 24% of identity in a 52% of the sequence, thus reaching a 39% of homology in this segment (Table B in S4 Table).

**Fig 1:**
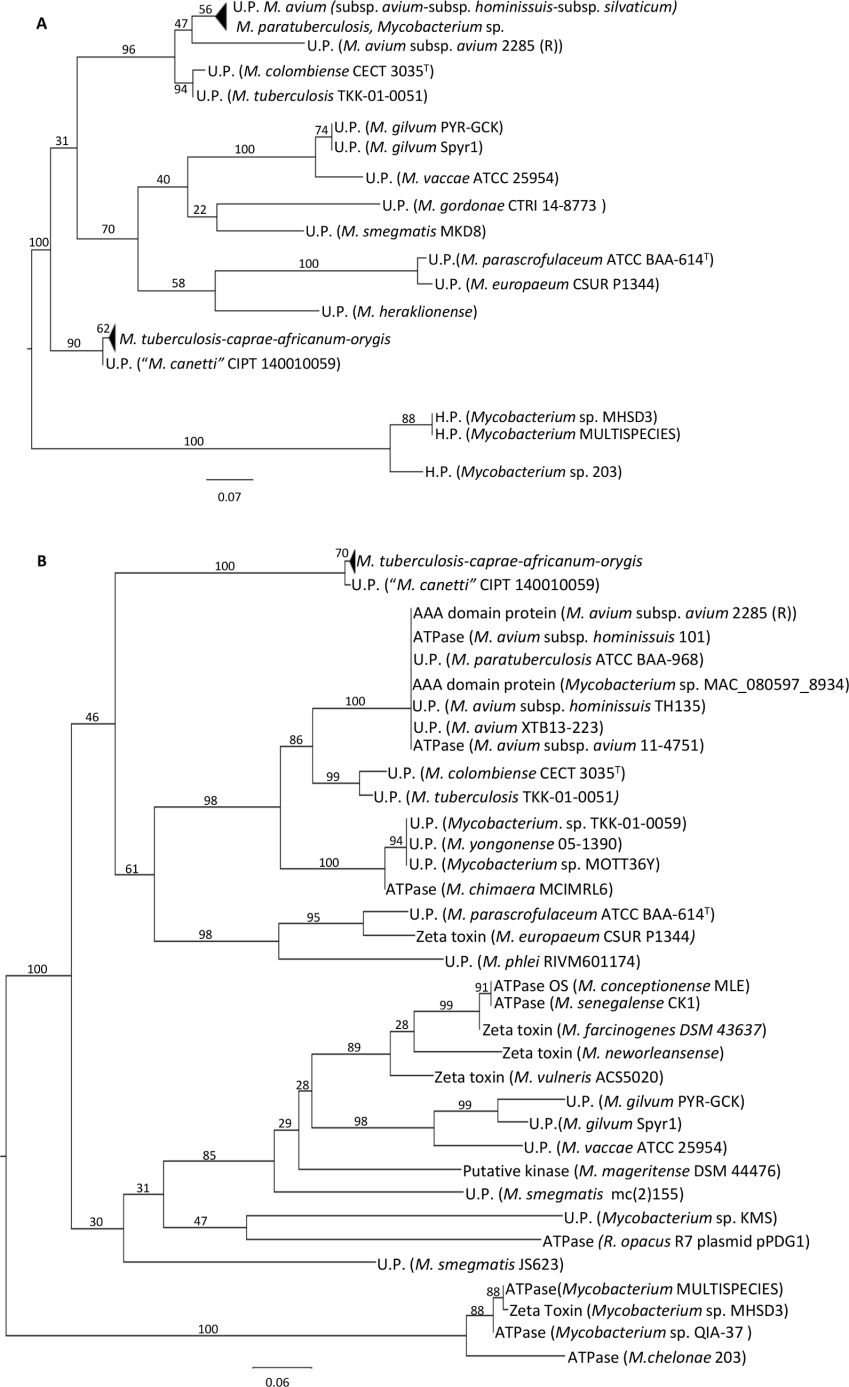
**ML trees of the H.P. (A) and toxin (B) with the most similar proteins from the databases (at least 50% identity covering at least 50% of the sequence)**. The tree is midpoint rooted.

BLAST analysis of the protein sequences identified similar proteins in other mycobacteria. The ML tree (100 bootstraps) generated by the closely related proteins (applying the criteria of at least 50% identity covering at least 50% of the sequence) present in the databases indicated that the proteins found in *Mycobacterium* sp. MHSD3 differ from the majority of proteins that are found in UniProt or GenBank (Fig 1A and 1B). Only a few proteins showing 100% coverage revealed more than 80% identity with the predicted TA system proteins of *Mycobacterium* sp. MHSD3. In the case of the H.P., the identities were higher than 92% and included a multispecies protein (WP_064408866.1) formed for the genomes of strains *Mycobacterium* sp. QIA-37 (accession number CP010071.1), *M*. *chelonae* strain 1558 (accession number JAOI01) (isolated from a sputum sample) and *M*. *chelonae* strain 15517 (accession number MLIR01, isolated from soft tissue). The identities of the zeta-toxin ranged between 88 and 99% with a multispecies ATPase (WP_070917639.1) from strains 1558 and 15517 (previously mentioned), and *M*. *chelonae* strain 15518 (accession number MLIS01, isolated from soft tissue); an ATPase from *Mycobacterium* sp. QIA-37 (WP_064408867.1); and a H.P. (WP_064408867) from *M*. *chelonae* 203 (accession number MLID01). The BLAST coverage results of the reminder of the proteins were over 90%, but exhibited identities less than 65% with proteins related to RGM and slow growing mycobacteria (SGM). These comparisons were repeated including the proteins of the TA systems from *S*. *pyogenes*, *S*. *pneumoniae* and *X*. *oryzae* pv. *oryzicola*, previously mentioned, with similar identity results against all mycobacterial proteins.

The H.P. of *Mycobacterium* sp. MHSD3 clustered with their closest relatives in a completely independent branch, with a bootstrap value (i.e., the number of times that a node or branch in a cluster analysis is recovered after randomly sampling the data a given number of times that the clustering process is repeated) of 100 (Fig 1A). The remainder of proteins shared less than 65% identify with those of strain *Mycobacterium* sp. MHSD3.

The zeta-toxin tree (Fig 1B) revealed two large branches supported by high bootstrap values. The second main branch of the tree, supported by a bootstrap value of 100, was only composed of the group of zeta-toxin/ATPases of *Mycobacterium* sp. MHSD3, which shared less than 65% identity with other proteins.

**Fig 2:**
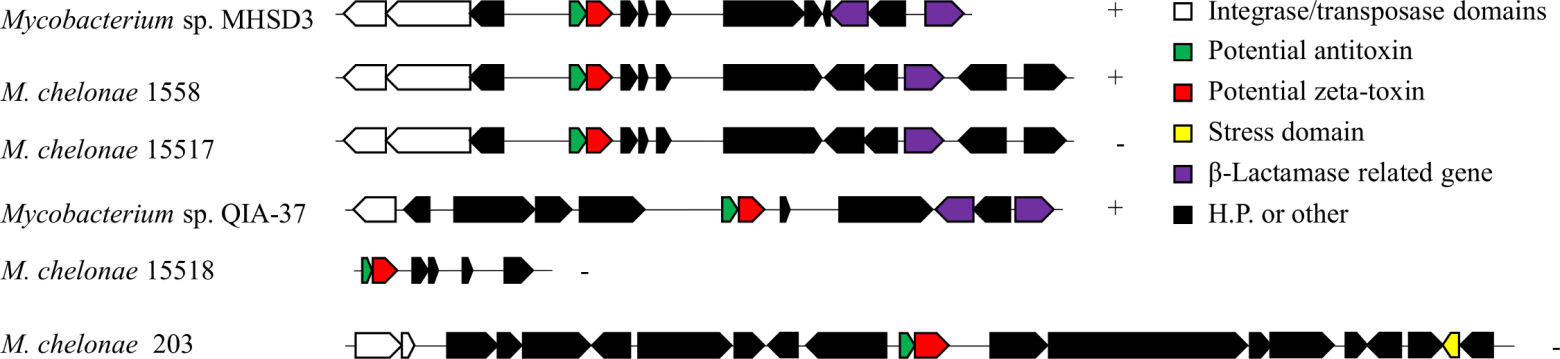
Synteny of the genes present in different strains encoding proteins with high identity to the protein sequences of *Mycobacterium* sp. MHSD3. The symbols +/- indicate the orientation in which the genes were found in the genomes.

Some synteny can be observed in the organization of these genes in the genomes in which the closest proteins are encoded (Fig 2). In all cases, genes encoding proteins with domains related to integrases or transposases were detected by Pfam and were located upstream of the hypothetical TA system. Genes related to β-lactamase activity were found in strains MHSD3, 1558, 15517 and QIA-37. Strain *M*. *chelonae* 203 showed a different organization; in this case, no β-lactamase-related genes were detected. Additionally, for this strain, Pfam detected a protein with a haem-degrading domain, which is related to hemin or peroxide-based stress (Fig 2). Not much information was obtained for *M*. *chelonae* strain 15518 since the organization shown in the figure was from a 3-kb contig. The synteny of the genes of interest is more conserved between strains MHSD3, 1558 and 15517 than the other proteins.

### Experimental test of the TA system functionality

The H.P. was cloned in the vector pBAD-HA, which promoter is inducible with arabinose; the hypothetical toxin was cloned in the vector pRSF-Duet, which promoter is inducible with IPTG. Hence, the expression of the H.P. was controlled by the presence of arabinose, while the expression of the putative zeta-toxin was regulated by the presence of IPTG. With the aim of demonstrating the toxic effect of the putative zeta-toxin and the neutralization capacity of the H.P. over this toxic effect, an experimental test was performed with one of the correct clones confirmed by Sanger sequencing (i.e., the insert was cloned in the correct open reading frame with the correct orientation and without mutations).The final objective of the experiment was to demonstrate that the product of the two genes found could behave as a real TA system, showing the effect on the bacterial growth when 1) there was no induction (Control), 2) when the expression of the toxin was induced alone (addition of IPTG), 3) when the antitoxin was induced alone (addition of arabinose) and when the expression of both genes were induced (addition of IPTG and arabinose).

**Fig 3:**
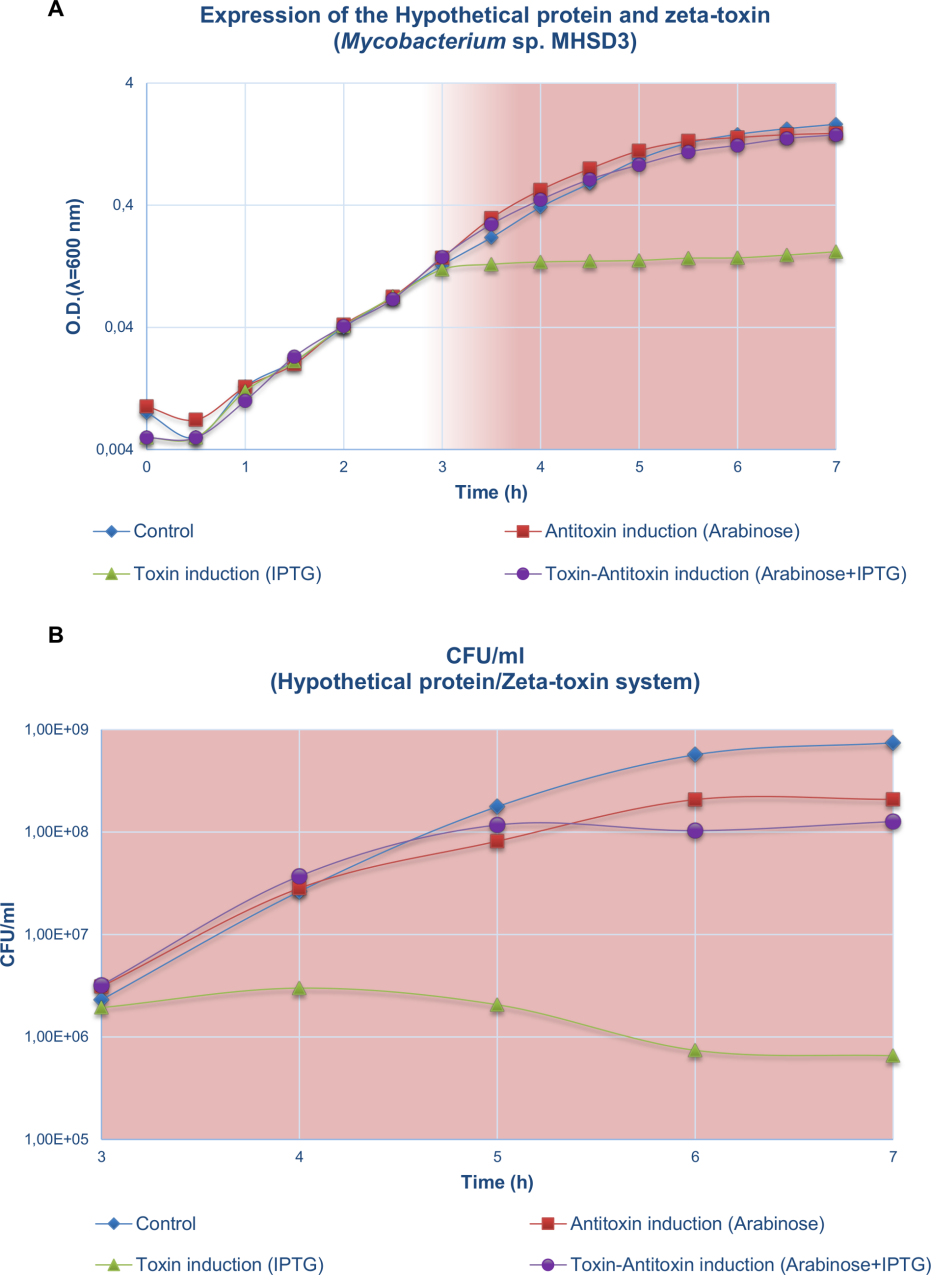
**Representation of a 7-hour growth curve (A) and the fate in terms of CFU/ml (B) from induction after 3 hours.** Differences between the control, induction of the antitoxin (Arabinose), induction of the toxin (IPTG), and induction of both elements (Arabinose and IPTG) can be observed in both cases. O.D. measurements at 600 nm with 1 ml of culture were done at intervals of 30 minutes, from the beginning to the end of the experiment. Colony forming units were counted each hour subsequently to the induction (3 h).

The fate of the four cultures was followed for 7 h. The resulting growth curves are shown in Fig 3A. This procedure was repeated three times to confirm the results (Figures A and B in S1 Fig Tables A, B and C in S1 Table). The control (without induction), the culture with the antitoxin induced, and the culture with both elements induced reached stationary phase, with slight differences observed between them, but with a clear tendency to grow even after induction for 3 h. The culture in which only the toxin was induced (with IPTG) reached the same level as the others during the first three hours; however, induction stopped its growth and it did not surpass an O.D. value of 0.3 at the end of the experiment (see S1 Table). This result was confirmed by viability counts (Fig 3B). All four conditions showed the same level of viable cells at the moment of induction (10^6^ CFU/ml). After induction, all cultures except for that with only the toxin induced continued to grow until 10^8^ CFU/ml. Induction of the toxin showed a clear decrease in CFU (to 10^5^ CFU/ml), indicating cell death.

### Experimental identification of the protein

The predicted theoretical molecular weights (MWs) of the H.P. and zeta-toxin obtained with pI/Mw were 13.12 and 21.64 kDa, respectively. This prediction was used to identify the potential bands corresponding to the toxin and antitoxin in the soluble fraction in polyacrylamide gels. Two over-expressed bands of the expected size were observed in cultures in which both elements were induced: one band between 20 and 25 kDa, representing the toxin, and another between 10 and 15 kDa, representing the H.P. (S2 Fig). The same banding pattern was obtained with additional replicates. No clear differences were detected in the other two cultures relative to the control. After induction, the immediate growth arrest or cell lysis due to the overproduction of the toxin could explain the disappearance of the corresponding band when only this element is induced. The largest instability of the antitoxin H.P. could result in a poor accumulation of the protein and, consequently, the particular band might not be seen.

The spectra obtained by MALDI-TOF MS did not match any of those in the Swiss-Prot database, and only one match to the same H.P. of *Mycobacterium* sp. QIA-37 was retrieved from GenBank; however, 13 peptides that matched the toxin and 9 that matched the H.P. were detected (Table 3 and 4), covering 80% and 65% of the toxin and H.P. sequence, respectively (S3 Fig), when searching the in-house database made from the theoretical protein sequences of the zeta-toxin and H.P. of *Mycobacterium* sp. MHSD3.

**Table 3:** Peptides identified matching the zeta-toxin sequence of *Mycobacterium* sp. MHSD3.

Start- End	Observed Mass	Expected Mass	Theoretical Mass	%^a^	M^b^	Peptide sequence
**85–89**	629.4743	628.4670	628.3908	0.0121	0	K.LDLIR.S
**52–57**	680.4117	679.4044	679.3289	0.0111	0	R.AYEAAR.I
**132–136**	694.4388	693.4315	693.3558	0.0109	1	R.ARYER.L
**58–63**	717.4772	716.4699	716.3817	0.0123	0	R.IAEQTR.Q
**90–99**	1081.6030	1080.5958	1080.5200	0.007	0	R.SAQAADYTVR.L
**43–51**	1082.5730	1081.5657	1081.4829	0.0077	0	R.WPDEDPAPR.A
**118–129**	1222.7630	1221.7557	1221.6353	0.0099	0	R.VEAGGHSVPIEK.I
**41–51**	1366.7608	1365.7535	1365.6425	0.0081	1	K.QRWPDEDPAPR.A
**100–113**	1622.1286	1621.1213	1620.9814	0.0086	0	R.LLVLLVPEELTVQR.V
**22–40**	2113.3526	2112.3453	2112.1255	0.0104	0	K.FLAPLLHESVFVNADEIAK.Q
**64–84**	2313.4832	2312.4759	2312.2277	0.0107	0	R.QALISQGRPFIAETVFSHPSK.L
**171–190**	2321.4734	2320.4661	2320.2117	0.011	0	R.GQVVGTLTWPQWTPAPLWQR.W
**137–163**	2854.7691	2853.7618	2853.4549	0.0108	0	R.LWPLVVDAIALADSSVVFDNSSEPGPR.V

^a^Percentage of error between the theoretical and the experimental mass.

^b^Number of missed cleavages (lysine or arginine not digested by the trypsin).

**Table 4:** Peptides identified matching the H.P. sequence of *Mycobacterium* sp. MHSD3.

Start-End	Observed Mass	Expected Mass	Theoretical Mass	%^a^	M^b^	Peptide sequence
**2–10**	982.6832	981.6759	981.5356	0.0143	0	M.AAPVDRPTR.V
**32–39**	1053.6712	1052.6639	1052.5152	0.0141	0	K.QQLDHWAR.L
**55–66**	1261.8439	1260.8366	1260.6496	0.0148	0	R.VEAALSGQLSMR.E
**88–98**	1266.8578	1265.8505	1265.6728	0.0140	0	R.IAATHLQDELR.A
**54–66**	1417.9621	1416.9548	1416.7507	0.0144	1	R.RVEAALSGQLSMR.E
**87–98**	1422.9743	1421.9670	1421.7739	0.0136	1	R.RIAATHLQDELR.A
**11–25**	1445.9502	1444.9430	1444.7158	0.0157	0	R.VASDLLDSAAAEGAR.Q
**53–66**	1574.0906	1573.0833	1572.8518	0.0147	2	R.RRVEAALSGQLSMR.E
**67–86**	2302.4850	2301.4777	2301.1376	0.0148	0	R.ELTPEEGVVFNAEIEVEL ER.R

^a^Percentage of error between the theoretical and the experimental mass.

^b^Number of missed cleavages (lysine or arginine not digested by the trypsin).

### Structure prediction

**Fig 4:**
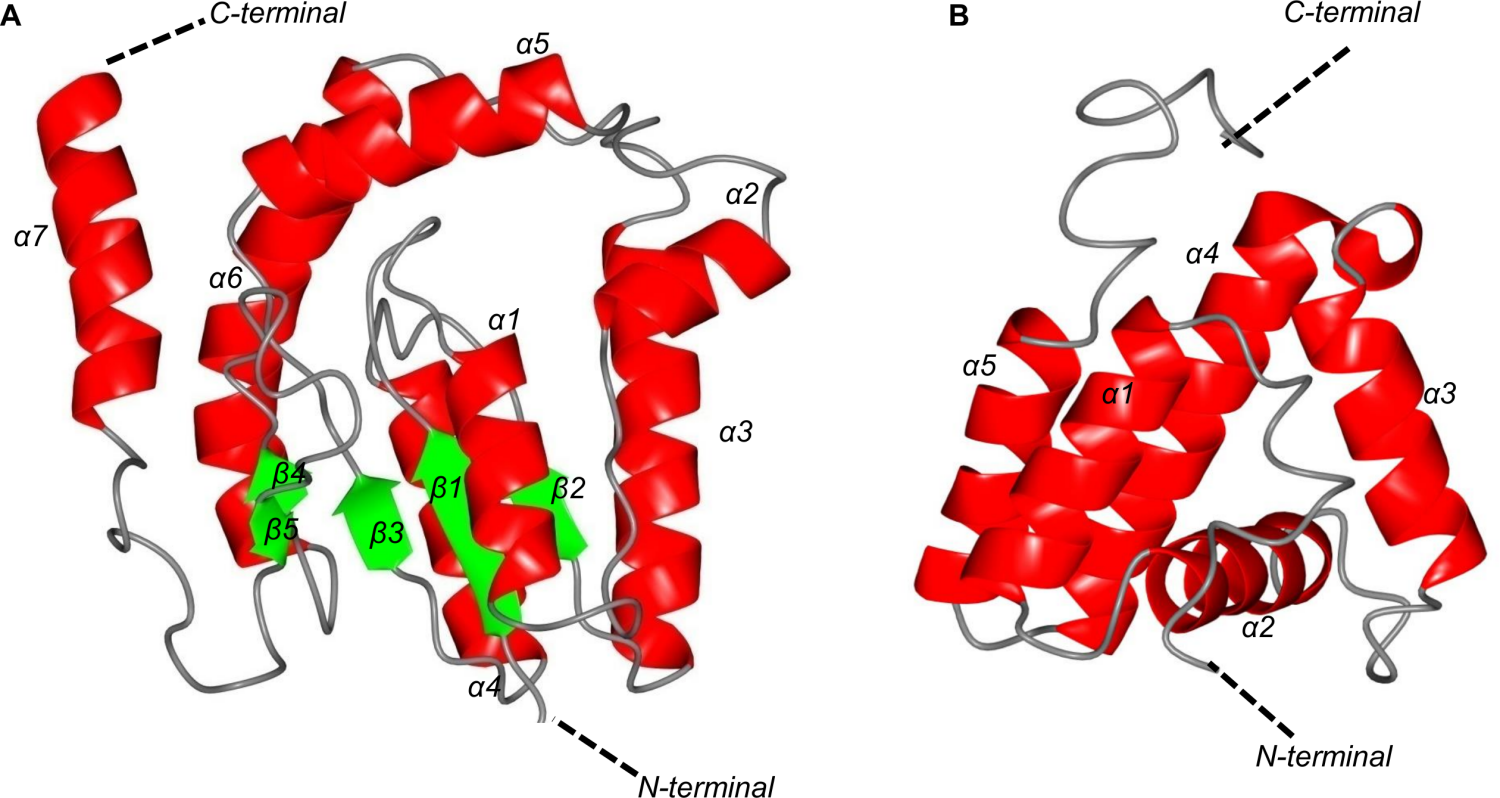
**Structural prediction of the zeta-toxin (A) and hypothetical protein (B) of strain *Mycobacterium* sp. MHSD3 by I-TASSER.** Protein sequences were submitted to I-TASSER for automatic structural prediction. Several templates were recruited from PDB to calculate the most optimal structure for each protein. For each protein, five models were obtained and the one showing the best statistical scores was selected.

The predicted structures of the zeta-toxin and H.P. obtained with I-TASSER software are shown in Fig 4A and 4B. The toxin was predicted to be composed of 7 α-helix and 5 β-layers, while the H.P. was predicted to be composed of 5 α-helices.

**Fig 5:**
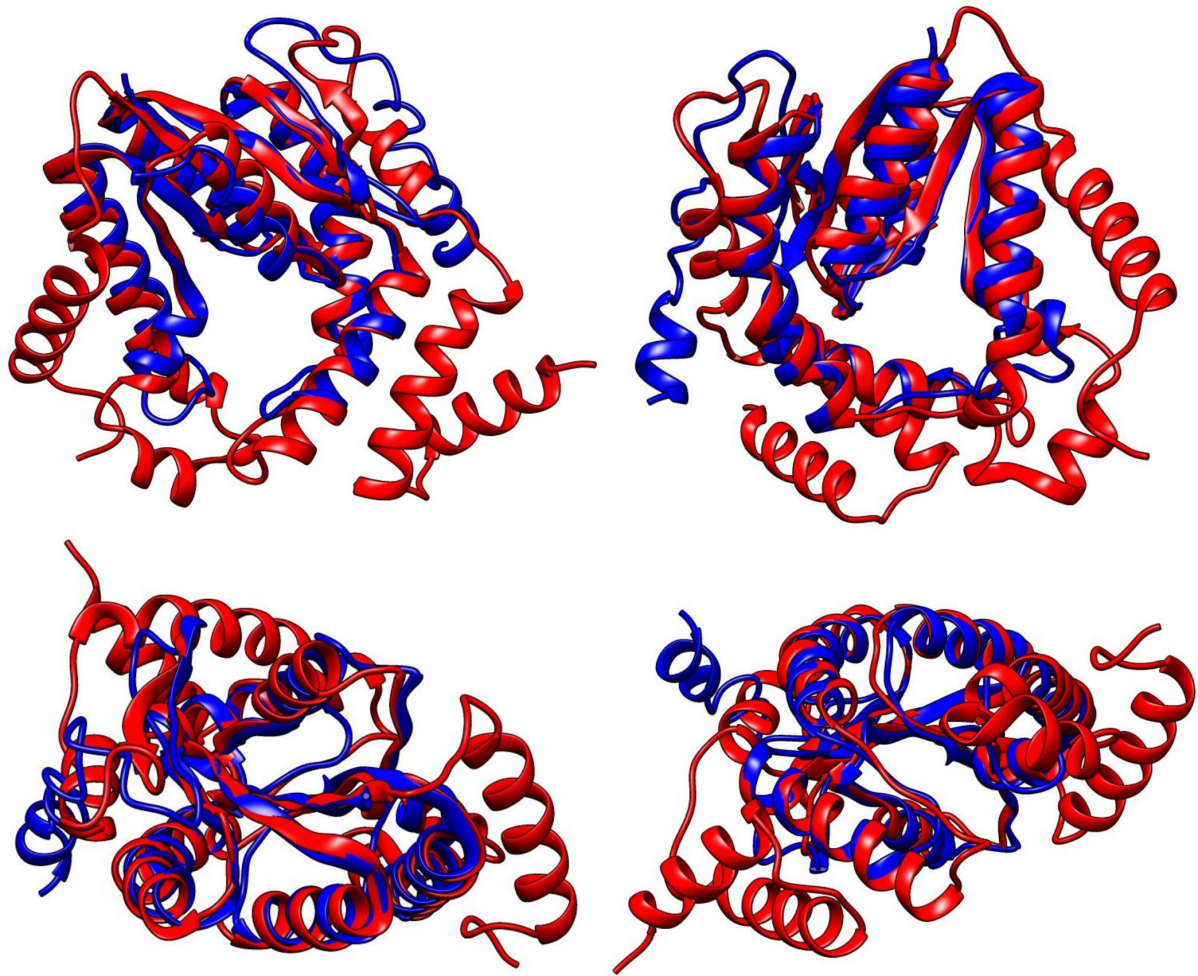
Structure superposition of the zeta-toxin present in plasmid pSM19035 of *S*. *pyogenes* and the toxin detected in the genome of strain *Mycobacterium* sp. MHSD3.

The best model for the H.P. exhibited a C-score of -3.85 and a TM-score of 0.30±0.10; the best model for the zeta-toxin exhibited a C-score of -0.59 and a TM-score of 0.64±0.13. From the available structures, the most suitable structural analogue identified by I-TASSER for the toxin was the ζ-toxin of the TA ε-*ζ* present in the plasmid pSM19035 of *S*. *pyogenes* (chain B of the complex with accession number 1GVN in RCSB PDB), supported by a TM-score of 0.811. Superposition of the two structures demonstrated a similar disposition of the secondary structural elements in three-dimensional space, at least with the nucleus of the structure encoded by the plasmid (Fig 5).

I-TASSER identified a potential ATP-binding site on the predicted structure with a C-score of 0.69 and identified 11 amino acids that are implicated in this ATP-binding site (S5 Table).

Conversely, the most suitable structural analogue for the H.P. according to I-TASSER was helicase PriA (accession number 4NL4 in PDB) of *Klebsiella pneumoniae*, a protein that has a longer sequence and is capable of binding to DNA but is not related to the TA system. The best model obtained had poor statistical values (C-score = -3.85, TM-score = 0.30±0.10). When superposed, the predicted structure of H.P. is matched with five alpha-helix situated between de DNA-binding domain and the helicase lobe 1 described in PriA (S4 Fig) [52]. The similarities between these two proteins likely only involve the DNA-binding domain included in the structure of the helicase PriA.

## Discussion

As mentioned above, epsilon/zeta systems are a type II TA system that exert a toxic effect throughout two mechanisms: phosphorylation of the peptidoglycan precursor UDP-N-acetylglucosamine (UNAG), forming UNAG-3P, an unreactive molecule that cannot be used by MurA, a key enzyme for peptidoglycan formation [20]; and phosphorylation of NAD/NAAD forming the unreactive substrates 3’-NADP/3’-NAADP [25]. The information exposed in this study does not allow to assign the TA system of *Mycobacterium* sp. MHSD3 to one of these mechanisms. Evidently, more in-depth studies are needed to explore and confirm this fact.

In the case of strain *Mycobacterium* sp. MHSD3, the TA system is encoded near an integrase and a transposase, which are located approximately 2-kb upstream, and near elements related to β-lactamases, downstream, forming a genomic block of approximately 10 kb. A similar organization is observed in other strains, such as *M*. *chelonae* 1558, *M*. *chelonae* 15517 and *Mycobacterium* sp. QIA-37. A different organization was detected in *M*. *chelonae* strain 203, in which no genes with domains related to β-lactamases were identified. Only one gene related to the response to stress conditions was observed. The transposase might be related to the TA system, representing a potential mobile genetic element. Nevertheless, deeper studies are needed to demonstrate if these elements are actually related, as well as demonstrate a possible relation with the antibiotic resistance or stress-response genes found.

Pfam is a domain database where families are groups of proteins defined by a Hidden Markov Model (HMM) [42]. Thus, families can be classified into superfamilies (Clans) which tend to be large and divergent groups of Pfam families related by sequence similarity, structure or HMM profile. Pfam detected domains in this H.P. gene related to the ParD-like family (ParD is the antitoxin of the TA system ParDE), but only based on 44% of the sequence coverage. Due to this result, deeper studies on the homology between H.P. and ParD were done, which proved that no homology relationships exist between them. Homology studies were also done using the three representative antitoxins of the three subfamilies (PezA, ε-antotixin and AvrRox2), obtaining the highest although not significative homology values with PezA of *S*. *pneumoniae* (33% identity on 17% of the sequence with 47% homology in this fragment). This result does not allow a conclusive classification of the H.P. into any of the sub-families of epsilon-zeta TA systems or assign a specific function. Nevertheless, the classification by Pfam in a superfamily mostly composed by representative of antitoxins of type II TA systems, along with the length and position of the gene that fits with the cognate element of the toxin, led to the selection of this gene to be tested as the potential antitoxin. An analysis of the amino acid sequence of the toxin concluded that the HMM model that best fits corresponds to the domain present in the AAA_33 family (which contains more than 5,000 proteins different in size and architecture). This family belongs to the superfamily P-Loop NTPases [53]. The toxin described here is not an AAA+ folded protein, but it contains a domain that Pfam considers similar to the one defined in the Pfam family AAA_33. All the members of this Pfam superfamily contain an ATP-binding site that was originally determined by phylogenetic analysis [54], which is currently known as the Walker-A motif, related to kinase functions [18,19]. This motif has also been predicted in the zeta-toxin of strain MHSD3, although the kinase activity needs to be experimentally tested. Homology studies with the representative toxins of the three sub-families (PezT, ζ-toxin and AvrRox1) showed the ζ-toxin as the most similar protein, with an identity of a 24% on a 52% of the overall sequence length, with a homology of 39% in this segment. In fact, the same protein was selected as a model for structure prediction by I-TASSER.

BLAST analysis of the respective protein sequences in both the GenBank and UniProt databases only showed a high percentage of coverage and identity with proteins annotated as ATPases (in the case of the toxin) and hypothetical proteins (in the case of the potential antitoxin) present in few strains of *M*. *chelonae*. These results, together with the represented trees, indicated the presence of proteins with a high degree of homology in *M*. *chelonae* strains.

A clear separation between SGM and RGM proteins (except for those of *M*. *phlei* and *M*. *vulneris)* was observed in the zeta-toxin tree, with the proteins of the MHSD3 group being completely separated from the rest. The same separation of the MHSD3 group was obtained in the case of the H.P. tree. High similarity was observed between the respective proteins present in MTC species (*M*. *tuberculosis*, *M*. *caprae*, *M*. *africanum* and *M*. *orygis*) and MAC species (with pathogens such as *M*. *avium* subsp. *sylvaticum* and *M*. *avium* subsp. *paratuberculosis*; and opportunistic pathogens such as *M*. *avium* subsp. *hominissuis*). These results indicate that this system is quite widespread in mycobacteria with pathogenic capacity. Together, these results indicate a new type II TA system that belongs to the group of epsilon/zeta systems.

Experimental induction of the toxin in *E*. *coli* BL21 cells showed a toxic effect on the cell population, indicating that the antitoxin, which is innocuous for the bacterial growth, can neutralize the toxic effect of zeta toxin, allowing the population to grow under these conditions. Confirmation of the proteins in cells by MALDI-TOF MS analysis was necessary to confirm the expression of the genes and to establish a possible correlation between these effects on the expression of the toxin and potential antitoxin. The detection of different bands present only in the culture in which both elements were expressed could be because they form a more stable complex when they interact, leading to a higher concentration of each protein at the end of the process. In fact, the lower stability of both kinds of proteins has been described in other epsilon/zeta toxins [55], and this could be the main explanation of why it is not possible to see their respective bands when only one component of the system is expressed. In the case of the toxin, another explanation could be that the exclusive expression of the toxin does not allow the synthesis of large amounts of biomass or induces cell lysis mainly due to overproduction of toxin, resulting in a lower amount of protein, and exclusive expression of the unstable antitoxin allows its faster degradation because it cannot interact with its cognate toxin.

The structural predictions by I-TASSER showed a zeta-toxin composed of 6 α-helices and 7 β-layers that fit into a tertiary structure similar to that of the ζ-toxin of the plasmid pSM19035 of *S*. *pyogenes*, a well-defined TA ε-ζ system [13,19], which is longer in sequence, but coincides with the positions of the 5 α-helices and 2 β-layers (TM-score = 0.811). The analysis also revealed the presence of a Walker-A motif, with a C-score = 0.609, in which 11 amino acids were implicated. A score higher than 0.5 indicates that most of these amino acids form a part of the ATP-binding site. Regarding the potential antitoxin, its structure was predicted to be composed of 5 α-helices, but no significant structural analogues were recognized. Nevertheless, these structural results are only predictions, and further studies are needed to determine the actual structures of the proteins of this module.

## Conclusions

A type II TA system was found in the clinical isolate *Mycobacterium* sp. MHSD3. The predicted structure of the toxin of this TA system coincides with the structure of the already defined ζ-toxin of the plasmid pSM19035. Although the toxin and its cognate antitoxin of the system appear to be quite different, the results indicate that the SGM and other RGM have potentially homologous proteins encoded in their genomes.

The experimental data shows the toxic effects of the toxin on a bacterial population when expressed alone and how the H.P., which overlaps upstream with the toxin gene, counteracts the toxic effects when expressed simultaneously with the toxin. This demonstrates that this H.P. acts as an antidote to the module and, for this reason, should be classified as the antitoxin of the TA system. Additional studies are needed to determine the mechanism of action of this particular TA system.

## Supporting information

S1 Table A) Optical density values at 600 nm of the four different conditions obtained for the first expression assay, B) the second expression assay and C) the third expression assay.
(PDF)

S2 Table List of protein sequences used in the phylogenetic tree of the H.P. Strains and accession numbers are included for each case.
(PDF)

S3 Table List of protein sequences used in the phylogenetic tree of the zeta-toxin. Strains and accession numbers are included for each case.
(PDF)

S4 Table **BLAST of the comparison of A) H.P. with ParD, ε-antitoxin, PezA and AvrRxo2 and B) the comparison of zeta-toxin with ζ-toxin, PezT and AvrRxo1.** The H.P. showed the highest result with PezA of *S*. *pneumoniae* according to the coverage, identity and homologous positions (Positives). No significant results were obtained with ε-antitoxin and AvrRxo2. The zeta-toxin showed the highest results with ζ-toxin of *S*. *pyogenes* according to the same parameters.
(PDF)

S5 Table List of amino acids involved in the predicted ATP-binding site made by I-TASSER in the zeta-toxin. (PDF)

S1 Fig **Representation of a 7-hour growth curve of the first (A) second (B) expression assays.** Evolution of the curves confirm de reproduction of the effect of the toxin on the cell population and the antitoxic activity of the H.P. Results are represented at logarithmic scale.
(TIFF)

S2 Fig Banding patterns obtained from protein extractions of the 7-h culture under four conditions. Protein extraction was performed by sonication from a 7 h-growth culture in the four induction conditions. A total volume of 20 μl of sample were loaded in each well of a polyacrylamide gel 4–20%. Gel was stained with Comassie blue. M indicates the molecular weight marker.
(TIFF)

S3 Fig Sequence covered by the peptides identified by MALDI-TOF MS analysis. Bands potentially corresponding to hypothetical protein and zeta-toxin were extracted from the polyacrylamide gel and analyzed by MALDI-TOF MS for protein identification. The sequence of the peptides obtained in each case were superposed with the original sequence of each protein. The sequence covered along the total sequence is shown in red, corresponding to 156 amino acids for the toxin and 78 for the hypothetical protein.
(TIFF)

S4 Fig Superposition of the tridimensional structures of H.P. and PriA. The α-helix of the H.P. are highlighted in green.
(TIFF)
