# Supplementary material for: First insights into a type II toxin-antitoxin system from the clinical isolate Mycobacterium sp. MHSD3, similar to epsilon/zeta systems
Source: PLoS One. 2017 Dec 13;12(12):e0189459. doi: 10.1371/journal.pone.0189459 (PMC5728571; doi:10.1371/journal.pone.0189459)
Supplement: S1 Table — A) Optical density values at 600 nm of the four different conditions obtained for the first expression assay, B) the second expression assay and C) the third expression assay. (PDF) [file pone.0189459.s001.pdf]

S1A Table. Optical density values at 600 nm of the four different conditions obtained for the first expression assay

Time (h)	Control	Antitoxin induction (Arabinose)	Toxin induction (IPTG)	Toxin-Antitoxin induction (Arabinose+IPTG)
0	0.001	0.001	0.001	0.001
0.5	0.002	0.002	0.001	0.002
1	0.012	0.011	0.011	0.01
1.5	0.027	0.028	0.028	0.025
2	0.055	0.054	0.049	0.049
2.5	0.117	0.119	0.112	0.112
3	0.241	0.282	0.193	0.258
3.5	0.462	0.521	0.224	0.496
4	0.814	0.857	0.241	0.775
4.5	1.2	1.191	0.243	1.065
5	1.414	1.42	0.256	1.271
5.5	1.574	1.532	0.249	1.427
6	1.784	1.586	0.265	1.561
6.5	1.862	1.615	0.273	1.626
7	1.936	1.633	0.273	1.702

S1B Table. Optical density values at 600 nm of the four different conditions obtained for the second expression assay

Time (h)	Control	Antitoxin induction (Arabinose)	Toxin induction (IPTG)	Toxin-Antitoxin induction (Arabinose+IPTG)
0	0.006	0.007	0.006	0.007
0.5	0.007	0.007	0.007	0.007
1	0.011	0.01	0.012	0.012
1.5	0.024	0.022	0.025	0.025
2	0.041	0.038	0.042	0.043
2.5	0.074	0.074	0.077	0.079
3	0.147	0.158	0.129	0.152
3.5	0.238	0.342	0.14	0.266
4	0.442	0.595	0.15	0.435
4.5	0.781	0.946	0.151	0.667
5	1.175	1.268	0.156	0.914
5.5	1.389	1.49	0.155	1.127
6	1.572	1.594	0.157	1.335
6.5	1.74	1.656	0.158	1.488
7	1.882	1.704	0.16	1.617

S1C Table. Optical density values at 600 nm of the four different conditions obtained for the third expression assay.

Time (h)	Control	Antitoxin induction (Arabinose)	Toxin induction (IPTG)	Toxin-Antitoxin induction (Arabinose+IPTG)
0	0.008	0.009	0.005	0.005
0.5	0.005	0.007	0.005	0.005
1	0.013	0.013	0.012	0.01
1.5	0.021	0.02	0.021	0.023
2	0.04	0.042	0.041	0.041
2.5	0.071	0.071	0.071	0.068
3	0.129	0.148	0.119	0.15
3.5	0.217	0.313	0.131	0.279
4	0.385	0.533	0.137	0.443
4.5	0.603	0.795	0.139	0.65
5	0.95	1.115	0.141	0.855
5.5	1.288	1.34	0.147	1.091
6	1.52	1.433	0.148	1.235
6.5	1.69	1.512	0.156	1.405
7	1.836	1.552	0.166	1.503
