# Supplementary material for: First insights into a type II toxin-antitoxin system from the clinical isolate Mycobacterium sp. MHSD3, similar to epsilon/zeta systems
Source: PLoS One. 2017 Dec 13;12(12):e0189459. doi: 10.1371/journal.pone.0189459 (PMC5728571; doi:10.1371/journal.pone.0189459)
Supplement: S2 Table — Strains and accession numbers are included for each case. (PDF) [file pone.0189459.s002.pdf]

**S2 Table. List of protein sequences used in the phylogenetic tree of the H.P**. Strains and accession numbers are included for each case.

| | Protein ID | Protein name |
|---|---|---|
| *M. canettii* CIPT 140010059 | WP_014000191.1 | hypothetical protein |
| *M. tuberculosis* Haarlem/NITR202 | AGL22129.1 | hypothetical protein |
| *M. orygis* 112400015 | EMT37385.1 | hypothetical protein |
| *M. bovis* BCG / Pasteur 1173P2 | CAL70390.1 | hypothetical protein |
| *M. bovis* ATCC BAA-935 / AF2122/97 | CDO41612.1 | hypothetical protein |
| *M. tuberculosis* ATCC 35801 / TMC 107 / Erdman | WP_003401860.1 | hypothetical protein |
| *M. africanum* GM041182 | WP_003401860.1 | hypothetical protein |
| *M. bovis* BCG-1 | WP_014000191.1 | hypothetical protein |
| *M. tuberculosis* ATCC 25618 / H37Rv | CCP43097.1 | hypothetical protein |
| *M. tuberculosis* MT43 | WP_003401860.1 | hypothetical protein |
| *M. tuberculosis* C | EAY58797.1 | hypothetical protein |
| *M. tuberculosis* BTB05-013 | KCN22862.1 | hypothetical protein |
| *M. tuberculosis* MAL010121 | WP_003401860.1 | hypothetical protein |
| *M. tuberculosis* OFXR-27 | WP_003401860.1 | hypothetical protein |
| *M. tuberculosis* M1034 | WP_003401860.1 | hypothetical protein |
| *M. tuberculosis* MD15956 | KAQ14453.1 | hypothetical protein |
| *M. tuberculosis* W-148 | WP_003401860.1 | hypothetical protein |
| *M. bovis* BCG 26 | AMC48905.1 | hypothetical protein |
| *M. colombiense* CECT 3035$^T$ | WP_007770931.1 | hypothetical protein |
| *M. tuberculosis* TKK-01-0051 | KBZ62316.1 | hypothetical protein |
| *M. avium* subsp. *paratuberculosis* 08-8281 | WP_003876750.1 | hypothetical protein |
| *M. avium* subsp. *avium* 2285 (R) | WP_009976725.1 | hypothetical protein |
| *M. avium* XTB13-223 | WP_009976725.1 | hypothetical protein |
| *M. avium* MAV_120709_2344 | WP_009976725.1 | hypothetical protein |
| *M. avium* subsp. *hominissuis* 101 | WP_009976725.1 | hypothetical protein |
| *M. avium* 05-4293 | ETA92680.1 | hypothetical protein |
| *M. avium* 10-5560 | ETB53215.1 | hypothetical protein |
| *M. avium* subsp. *hominissuis* TH135 | WP_009976725.1 | hypothetical protein |
| *M. avium* subsp. *avium* 11-4751 | ETB21122.1 | hypothetical protein |
| *M. avium* subsp. *hominissuis* 100 | WP_009976725.1 | hypothetical protein |
| *M. avium* subsp. *avium* 10-9275 | WP_009976725.1 | hypothetical protein |
| *M. avium* subsp. *silvaticum* ATCC 49884 | ETB09947.1 | hypothetical protein |
| *M. avium* 10-5581 | ETA97490.1 | hypothetical protein |
| *M. avium* 104 | ABK67732.1 | hypothetical protein |
| *Mycobacterium* sp. MAC_080597_8934 | WP_033710872.1 | hypothetical protein |
| *M. paratuberculosis* ATCC BAA-968 / K-10 | AAS04094.1 | hypothetical protein |
| *M. avium* subsp. *hominissuis* A5 | KDO96018.1 | hypothetical protein |
| *M. avium* subsp. *paratuberculosis* 10-5864 | ETB03997.1 | hypothetical protein |
| *M. avium* subsp. *paratuberculosis* 10-4404 | WP_003878042.1 | hypothetical protein |
| *M. avium* subsp. *hominissuis* 10-5606 | ETB41458.1 | hypothetical protein |
| *M. tuberculosis* | WP_031730560.1 | hypothetical protein |
| *M. parascrofulaceum* ATCC BAA-614 | EFG79139.1 | hypothetical protein |

| | | |
|---|---|---|
| *M. heraklionense* | WP_047317834.1 | hypothetical protein |
| *M. vaccae* ATCC 25954 | WP_003929789.1 | hypothetical protein |
| *M. europaeum* CSUR P1344 | CQD16798.1 | hypothetical protein |
| *M. gordonae* CTRI 14-8773 | WP_055576679.1 | hypothetical protein |
| *M. gilvum* PYR-GCK | ABP47663.1 | hypothetical protein |
| *M. gilvum* DSM 45189 / LMG 24558 / Spyr1 | ADU01179.1 | hypothetical protein |
| *M. smegmatis* MKD8 | WP_003894879.1 | hypothetical protein |
| *M. avium* subsp. *avium* 2285 R | EUA33059.1 | hypothetical protein |
| *M. chelonae* | WP_064408866.1 | hypothetical protein |
| *M. chelonae* 203 | OHT78135.1 | hypothetical protein |
