# Supplementary material for: First insights into a type II toxin-antitoxin system from the clinical isolate Mycobacterium sp. MHSD3, similar to epsilon/zeta systems
Source: PLoS One. 2017 Dec 13;12(12):e0189459. doi: 10.1371/journal.pone.0189459 (PMC5728571; doi:10.1371/journal.pone.0189459)
Supplement: S3 Table — Strains and accession numbers are included for each case. (PDF) [file pone.0189459.s003.pdf]

S3 Table. List of protein sequences used in the phylogenetic tree of the zeta-toxin. Strains and accession numbers are included for each case.

	Protein ID	Protein name
M. parascrofulaceum ATCC BAA-614	EFG79138.1	Hypothetical protein
Mycobacterium sp. KMS	WP_011560054.1	ATPase
M. europaeum CSUR P1344	CQD16793.1	Zeta toxin
Mycobacterium sp. JS623	AGB26847.1	hypothetical protein plasmid
M. orygis 112400015	EMT37384.1	hypothetical protein
M. bovis BCG / Pasteur 1173P2	CAL70389.1	hypothetical protein
M. tuberculosis CDC 1551 / Oshkosh	WP_003401859.1	hypothetical protein
M. bovis ATCC BAA-935 / AF2122/97	CDO41611.1	hypothetical protein
M. tuberculosis ATCC 35801 / TMC 107 / Erdman	WP_003401859.1	hypothetical protein
M. tuberculosis ATCC 25177 / H37Ra	WP_003401859.1	hypothetical protein
M. africanum GM041182	WP_003401859.1	hypothetical protein
M. caprae MB2	WP_003401859.1	hypothetical protein
M. bovis 1595	AKQ99931.1	ATPase
M. tuberculosis ATCC 25618 / H37Rv	CCP43096.1	hypothetical protein
M. tuberculosis MT43	WP_003401859.1	hypothetical protein
M. tuberculosis C	EAY58796.1	hypothetical protein
M. tuberculosis BTB05-013	KCN22861.1	hypothetical protein
M. tuberculosis MAL010121	WP_003401859	hypothetical protein
M. tuberculosis OFXR-27	WP_003401859	hypothetical protein
M. tuberculosis M1034	WP_003401859	hypothetical protein
M. tuberculosis MD15956	KAQ14452.1	hypothetical protein
M. tuberculosis W-148	WP_003401859	hypothetical protein
M. bovis BCG 26	AMC48904.1	hypothetical protein
M. canettii CIPT 140010059	WP_014000190	ATPase
M. mageritense DSM 44476 = CIP 104973	WP_036442085	ATPase
M. tuberculosis	COV58376.1	ZTL protein
M. chimaera MCIMRL6	WP_054585353	ATPase
Mycobacterium sp. 05-1390	WP_014711570	ATPase
Mycobacterium sp. TKK-01-0059	WP_014711570	ATPase
Mycobacterium sp. MOTT36Y	WP_014711570	ATPase
M. phlei RIVM601174	WP_003888374	ATPase
M. tuberculosis TKK-01-0051	WP_044485837	ATPase
M. vaccae ATCC 25954	WP_003929790	ATPase
M. colombiense CECT 3035^{T}	WP_007770932	ATPase
M. conceptionense MLE	KMV19162.1	ATPase
M. senegalense CK1	WP_019346252	ATPase
Rhodococcus opacus R7 plasmid pPDG1	AII10527.1	ATPase plasmid
M. farcinogenes DSM 43637	WP_036387328	ATPase
M. vulneris ACS5020	OCB45067.1	ATPase
M. gilvum DSM 45189 / LMG 24558 / Spyr1	WP_013472824	ATPase
M. neworleansense	CRZ14295.1	Zeta toxin
M. gilvum PYR-GCK	WP_011896020	ATPase
M. smegmatis ATCC 700084 / mc2155	YP_887740.1	ATPase

Mycobacterium. sp. MAC_080597_8934	WP_003876749	ATPase
M. paratuberculosis ATCC BAA-968 / K-10	AAS04093.1	ATPase
M. avium subsp. hominissuis 101	WP_003876749.1	ATPase
M. avium subsp. *avium* 2285 R	WP_003876749.1	ATPase
M. avium XTB13-223	WP_003876749.1	ATPase
M. avium subsp. *hominissuis* TH135	WP_003876749.1	ATPase
M. avium subsp. *avium* 11-4751	ETB21123.1	ATPase
M. chelonae 1558	WP_070917639.1	ATPase
M. chelonae 15517	WP_070917639.1	ATPase
M. chelonae 15518	WP_070917639.1	ATPase
M. chelonae 203	WP_070921490.1	hypothetical protein
Mycobacterium sp. QIA-37	WP_064408867.1	ATPase
