# Supplementary material for: First insights into a type II toxin-antitoxin system from the clinical isolate Mycobacterium sp. MHSD3, similar to epsilon/zeta systems
Source: PLoS One. 2017 Dec 13;12(12):e0189459. doi: 10.1371/journal.pone.0189459 (PMC5728571; doi:10.1371/journal.pone.0189459)
Supplement: S4 Table — BLAST of the comparison of A) H.P. with ParD, ε-antitoxin, PezA and AvrRxo2 and B) the comparison of zeta-toxin with ζ-toxin, PezT and AvrRxo1. The H.P. showed the highest result with PezA of S. pneumoniae according to the coverage, identity and homologous positions (Positives). No significant results were obtained with ε-antitoxin and AvrRxo2. The zeta-toxin showed the highest results with ζ-toxin of S. pyogenes according to the same parameters. (PDF) [file pone.0189459.s004.pdf]

S4A Table. BLAST of the comparison of H.P. with ParD, ε-antitoxin, PezA and AvrRxo2. Highest results were shown with PezA of *S. pneumoniae* according to the coverage, identity and homologous positions (Positives). No significant results were obtained with ε-antitoxin and AvrRxo2.

	**Query cover**	**Identity**	**Positives**	**Gaps**
ε-antitoxin (*S. pyogenes*)	No significative	No significative	///	///
PezA (*S. pneumoniae*)	17%	33%	10/21(47%)	0/21(0%)
AvrRxo2 (*Xanthomonas*)	No significative	No significative	///	///
ParD (*E. coli*)	5%	44%	7/9(77%)	2/9(22%)

S4B Table. BLAST of the comparison of zeta-toxin with ζ-toxin, PezT and AvrRxo1. Highest results were shown with ζ-toxin of *S. pyogenes* according to the coverage, identity and homologous positions (Positives).

	**Query cover**	**Identity**	**Positives**	**Gaps**
ζ-toxin (*S. pyogenes*)	52%	24%	45/113(39%)	12/113(10%)
PezT (*S. pneumoniae*)	6%	58%	10/12(83%)	0/12(0%)
AvrRxo1 (*Xanthomonas*)	8%	44%	10/16(62%)	0/16(0%)
