# Supplementary material for: First insights into a type II toxin-antitoxin system from the clinical isolate Mycobacterium sp. MHSD3, similar to epsilon/zeta systems
Source: PLoS One. 2017 Dec 13;12(12):e0189459. doi: 10.1371/journal.pone.0189459 (PMC5728571; doi:10.1371/journal.pone.0189459)
Supplement: S5 Table — (PDF) [file pone.0189459.s005.pdf]

S5 Table. List of amino acids involved in the predicted ATP-binding site made by I-TASSER in the zeta-toxin.

C-score	Cluster size	PDB Hit	Lig Name	Ligand Binding Site Residues
0.69	213	1ko5B	ATP	11,12,13,14,15,16,17,113,156,159,161
