# Supplementary figures and images for: First insights into a type II toxin-antitoxin system from the clinical isolate Mycobacterium sp. MHSD3, similar to epsilon/zeta systems
Source: PLoS One. 2017 Dec 13;12(12):e0189459. doi: 10.1371/journal.pone.0189459 (PMC5728571; doi:10.1371/journal.pone.0189459)

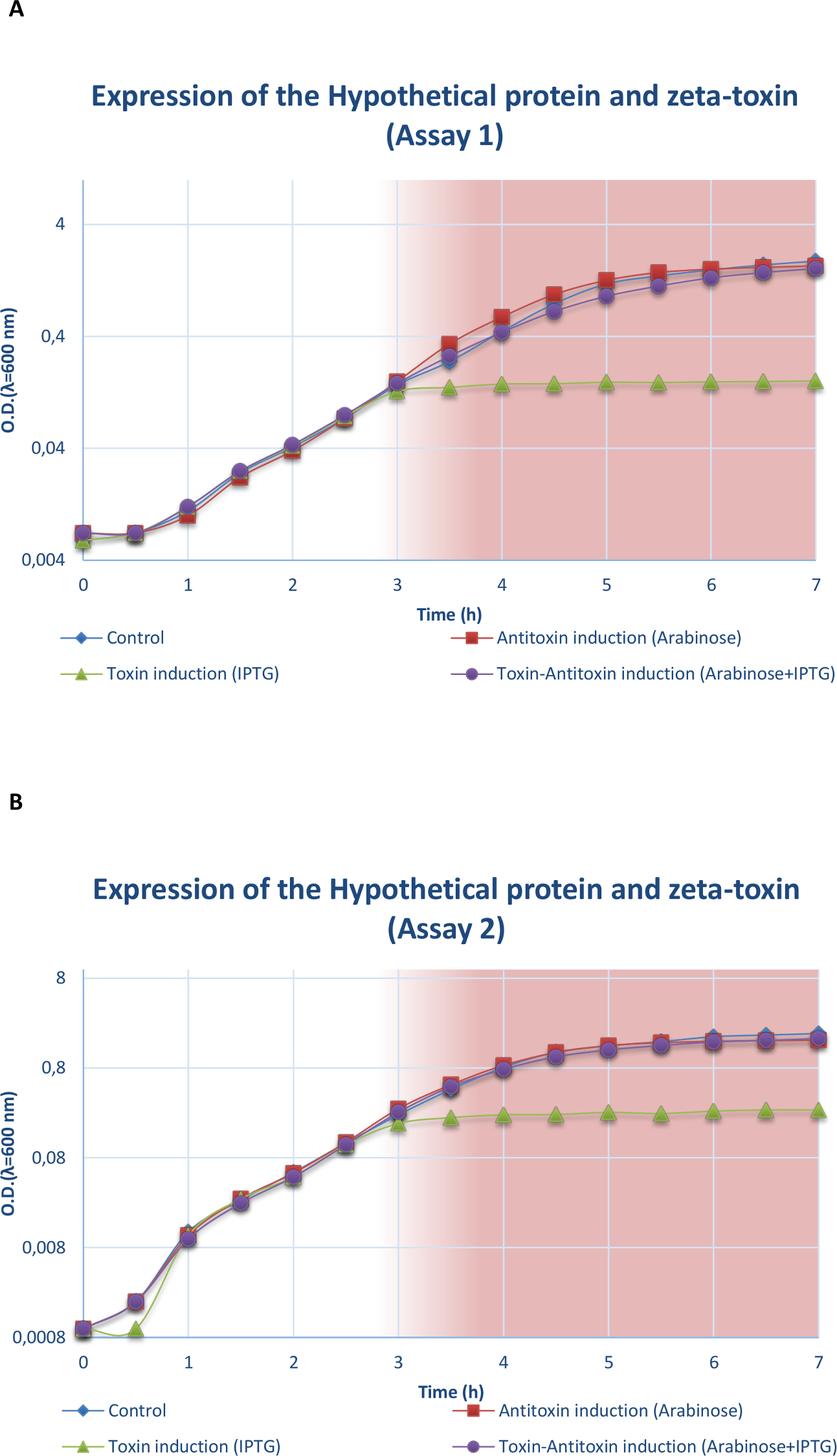

Supplement: S1 Fig — Representation of a 7-hour growth curve of the first (A) second (B) expression assays. Evolution of the curves confirm de reproduction of the effect of the toxin on the cell population and the antitoxic activity of the H.P. Results are represented at logarithmic scale. (TIFF) [file pone.0189459.s006.tiff]

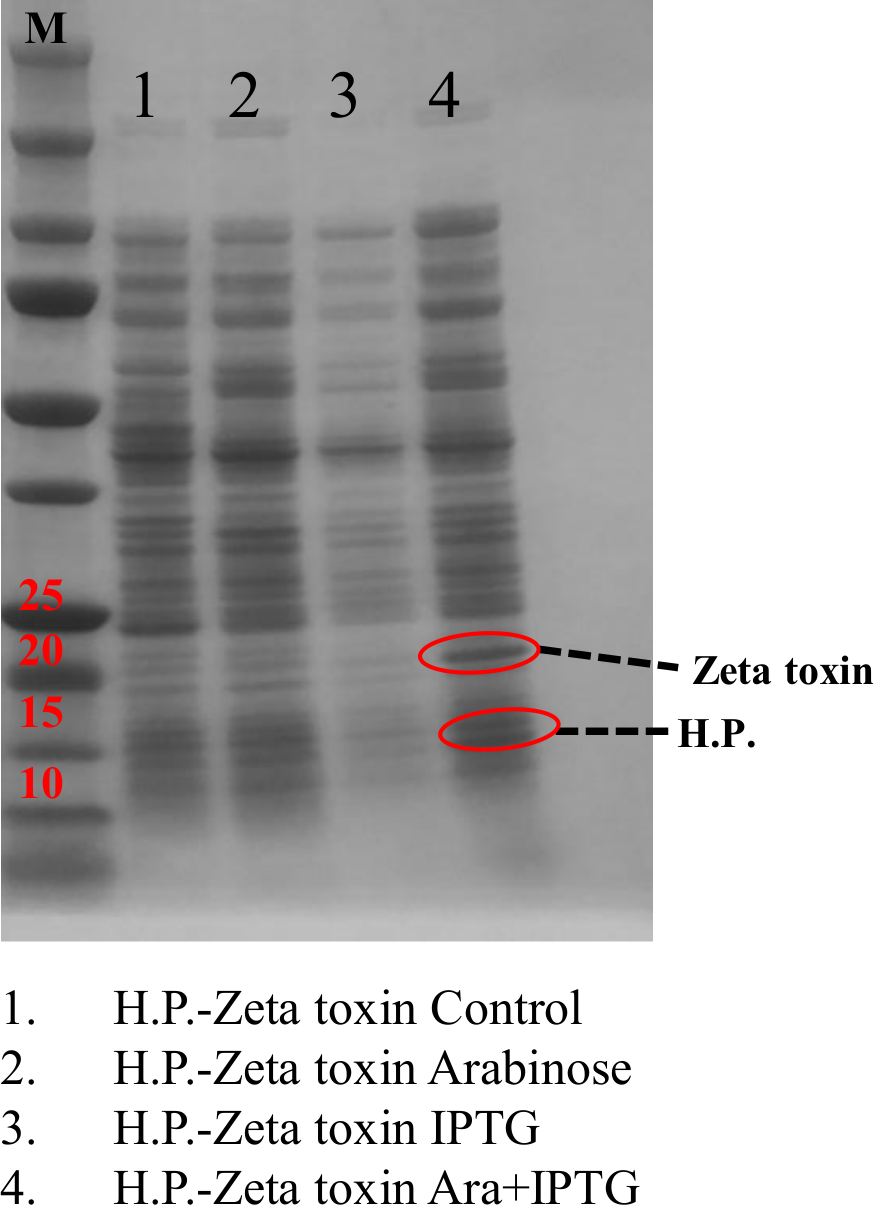

Supplement: S2 Fig — Protein extraction was performed by sonication from a 7 h-growth culture in the four induction conditions. A total volume of 20 μl of sample were loaded in each well of a polyacrylamide gel 4–20%. Gel was stained with Comassie blue. M indicates the molecular weight marker. (TIFF) [file pone.0189459.s007.tiff]

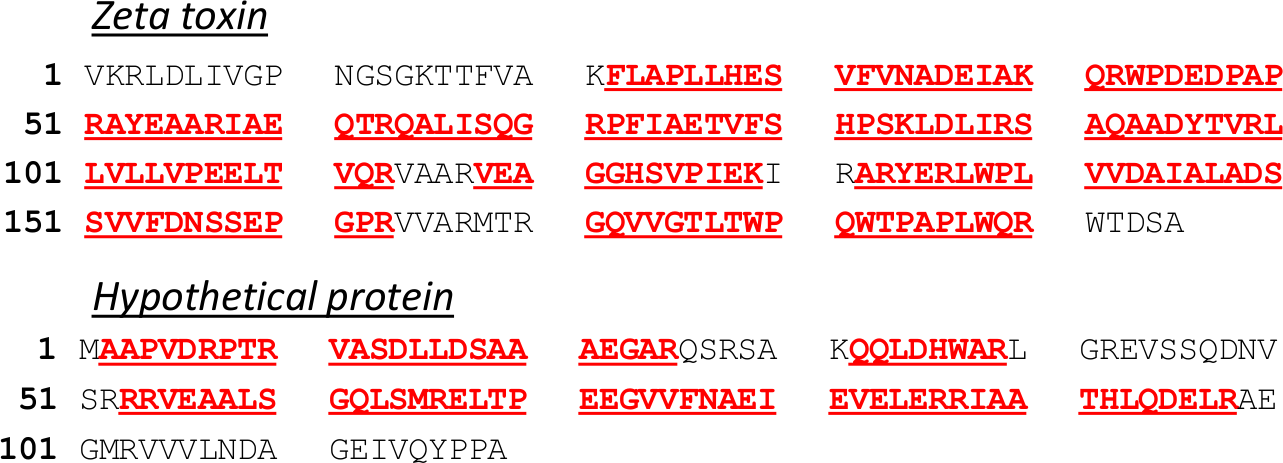

Supplement: S3 Fig — Bands potentially corresponding to hypothetical protein and zeta-toxin were extracted from the polyacrylamide gel and analyzed by MALDI-TOF MS for protein identification. The sequence of the peptides obtained in each case were superposed with the original sequence of each protein. The sequence covered along the total sequence is shown in red, corresponding to 156 amino acids for the toxin and 78 for the hypothetical protein. (TIFF) [file pone.0189459.s008.tiff]

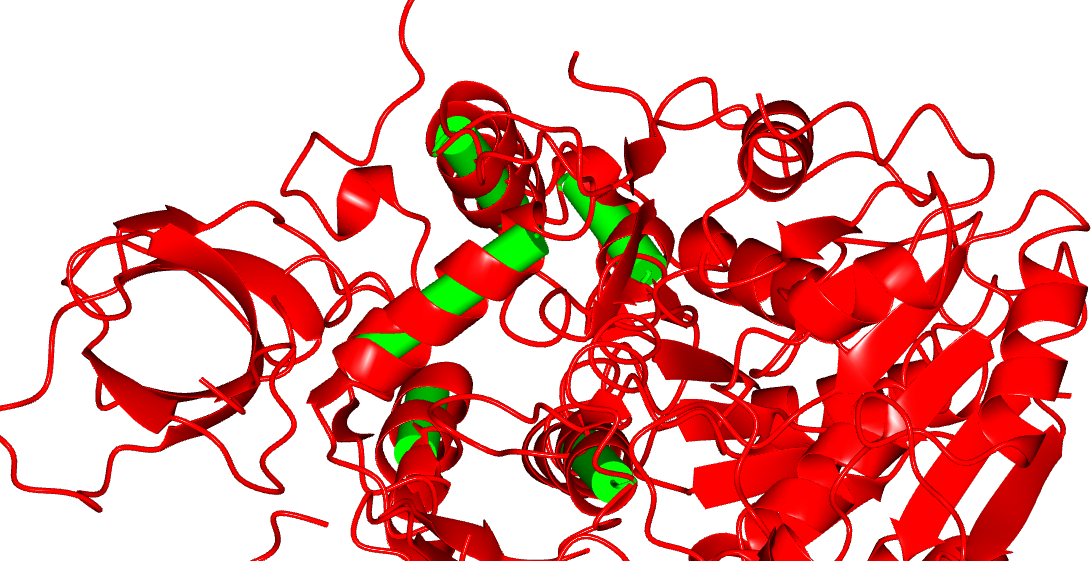

Supplement: S4 Fig — The α-helix of the H.P. are highlighted in green. (TIFF) [file pone.0189459.s009.tiff]
